# The cause of anorexia and proportion of its recovery in older adults without underlying disease: Results of a retrospective study

**DOI:** 10.1371/journal.pone.0224354

**Published:** 2019-10-24

**Authors:** Nobuyuki Maki, Eiji Nakatani, Toshiyuki Ojima, Tomoka Nagashima, Takane Harada, Fumiko Koike, Naoki Tosaka, Hiroshi Yoshida, Toshio Shimada

**Affiliations:** 1 Department of Emergency Medicine, Shizuoka General Hospital, Shizuoka City, Shizuoka, Japan; 2 Division of Statistical Analysis, Research Support Center, Shizuoka General Hospital, Shizuoka City, Shizuoka, Japan; 3 Division of Medical Statistics, Translational Research Center for Medical Innovation on Foundation for Biomedical Research and Innovation at Kobe, Kobe City, Hyogo, Japan; 4 Department of Community Health and Preventive Medicine, Hamamatsu University School of Medicine, Hamamatsu City, Shizuoka, Japan; 5 Community Medicine Network Center, Shizuoka General Hospital, Shizuoka City, Shizuoka, Japan; 6 Clinical Research Center, Shizuoka General Hospital, Shizuoka City, Shizuoka, Japan; University of Auckland, NEW ZEALAND

## Abstract

**Aim:**

It is difficult to determine whether or not end-of-life care is necessary for frail older adults complaining of anorexia without underlying disease, such as cancer or organ failure. The main reason for this is the lack of the specification of the anorexia cause and no understanding of the cause-providing factor and the prognostic factor. This study aimed to clarify the cause of anorexia, and the determinant of the cause and recovery from anorexia.

**Methods:**

Retrospective chart reviews were conducted on patients with anorexia without an underlying disease who were aged ≥65 years and visited the emergency department of a single tertiary care center between 2016 and 2017. Patient characteristics at hospital visit, the cause of anorexia, and diagnostic modalities were summarized. The diagnosis-providing rate, recovery rate, and the association between them were analyzed.

**Results:**

Eighty-three patients (mean age 82.3 years; 50.6% male) were investigated. In 67 patients (81%), the causes of anorexia were identified, including 18 patients (22%) with infection, 13 (16%) with benign gastrointestinal diseases, and 7 (8%) with cardiovascular diseases. In 16 patients (19%), the causes of anorexia were not identified despite examinations. The modality that most contributed to diagnosis was plain computed tomography followed by blood tests. The value regarding information in history-taking and physical examinations was limited. Sixty-five patients (78%) recovered. Only 73% of patients with a definite cause recovered; all patients with an unknown cause recovered.

**Conclusions:**

Older adults with anorexia are not always at the end of life, and efforts to identify the cause are crucial. Moreover, it is vital to realize the limitations associated with the treatment of infections and cardiovascular diseases.

## Introduction

Decision-making with regards to end-of-life care for older adults is an important and challenging process in developed countries [1]. Forty-three percent of all older adults aged ≥60 years require decision-making on end-of-life care [2]. This is often difficult because of several factors; we showed that one of the reasons is a lack of adequate data to predict the prognosis accurately [3]. In both the patients’ own and surrogate decision-making, preferences for or against treatments are greatly affected by the predicted prognosis [4, 5]. However, the prediction of the prognosis provided by medical doctors remains inaccurate [6, 7]. Furthermore, several predicting factors associated with prognosis have been found in patients with underlying diseases, such as malignancy [8] and heart failure [9]. However, prediction of prognosis is particularly difficult in frail older adults without underlying diseases because they already have low-level function at baseline, and even minor physical events can be fatal [10].

Anorexia often appears at the end of life [11] and is a powerful predictor of quality of life, morbidity, and mortality [12]. Although aging, acute and chronic diseases, and treatments are reported as anorexia causes [12], it is often difficult to differentiate between these causes. The determinant for providing any cause for anorexia is also not clear.

In Japan, older adults who are dying or die of anorexia without any cause are often considered to have senility, the terminal state of anorexia of aging [13]. Senility was the fifth leading cause of death in Japan in 2016; the number of cases had doubled from 2010 [14]. However, most patients who were diagnosed with senility were found to have a specific cause of death at autopsy [15]. Thus, there might be more unknown causes of death that are actually associated with anorexia than was identified previously. While anorexia of aging is frequently erroneously attributed to a normal part of the aging process [16], identifying the cause of anorexia in older adults without underlying diseases is not only useful for predicting prognosis, but also for identifying some specific causes that could be treatable. Accordingly, we hypothesized that the cause-specification in older adults with anorexia is important for planning therapy and predicting the prognosis.

Accordingly, the identification of cause and cause-providing factors for anorexia is important in frail older adults without underlying diseases. Thus, we studied the causes of anorexia and the determinant of the causes in frail older adults without underlying diseases. Furthermore, in these patients, the prognostic factors were also investigated.

## Methods

### Patients

The patients in this study were older adults aged 65 years or older who visited the emergency department of a single 720-bed tertiary care center from April 1, 2016, to March 31, 2017, and for whom symptoms such as “anorexia,” “hard to eat,” and “decreased oral intake” were documented as the chief complaints or present history in their medical records. These symptoms were usually reported by the patients or caregivers on the day of visiting the emergency department. We defined the day that they noticed these symptoms for the first time as the day of onset. Patients who met at least one of the following criteria were excluded: 1) patients who had been diagnosed with underlying diseases that could cause anorexia (such as malignancy and organ failure) before the emergency department visit, and 2) patients who had two or more episodes of anorexia in the study period. However, because 5 years is considered long-term survival in some malignancies (such as gastric cancer and colon cancer) [17, 18], we did not exclude patients with these malignancies who had undergone curative resection and were recurrence-free for 5 years. We incorporated only the first episode in patients with two or more anorexic episodes in the study period.

### Study design and data

We retrospectively investigated the medical records of the study patients between May 1 and June 30, 2017. Collected data included patient characteristics, such as age, sex, type of residence before the emergency department visit (home, facilities, etc.), ambulation at the onset of anorexia, number of days from the onset to the visit, past history, concomitant symptoms, diagnosis, with or without hospitalization, diagnostic modalities (history-taking, physical examination, blood tests, urinalysis, culture tests, radiography, plain computed tomography (CT), electrocardiography, enhanced CT, fiber endoscopy, and others), laboratory data, treatments, and prognosis. Concomitant symptoms were extracted from the medical records documented by the doctors; all patients who received an evaluation of unconsciousness were unconscious when they arrived at the emergency department. Doctors in charge or caregivers observed the amount of oral intake of the patients, and requirements for enteral or parenteral nutrition were determined by the doctors after visiting the emergency department. The prognosis was regarded as “recovered” if the patients recovered their oral intake and doctors evaluated that enteral and parenteral nutrition was not needed or could be ceased, “not changed” if enteral or parenteral nutrition was continued or followed conservatively with an expectation that death in the near feature was inevitable, or “dead” if the patients did not recover their oral intake and died. Moreover, the prognosis was decided based on whether the patients recovered or not. Whenever data on whether patients recovered or died could not be obtained through medical records, we telephoned the patients, their families, or, if they provided consent, the facilities they resided in, and we asked them about the prognosis, between August and December 2017.

We confirmed the patients’ diagnoses based on the description in the medical records. Malnutrition, hypovolemia, dehydration, and (prerenal) acute kidney injury were regarded not as causes of anorexia, but as results of anorexia [19,20]. Electrolyte abnormality was regarded as a cause of anorexia only if other causes (such as the presence of adverse drug effects) were supposed to be related to anorexia; otherwise, it was regarded as a result of anorexia [21]. We determined the cause of anorexia to be unknown if the diagnosis was unclear except the above; we determined the cause of anorexia be identified if other diagnoses existed. Diagnostic modalities were evaluated by what modalities were used and contributed to the diagnosis (showing evidence of a definite diagnosis or the need for further examination or admission). Treatments were evaluated as “performed” if radical treatments for the cause of anorexia, such as surgery for malignancy, percutaneous coronary intervention for acute coronary syndrome, and administration of antibiotics for infection, had been performed; these treatments did not include hydration or nutritional support. Two experienced physicians (N.M. and T.H.) independently determined through medical records whether or not the cause of anorexia had been proven, as well as what diagnostic modalities were used and contributed to the diagnosis and whether the cause of anorexia was treated or not. After that, they cross-checked the results, and in cases that were not confirmed, we finally determined after a joint discussion.

### Outcome

The primary outcome of this study was the cause of anorexia. The secondary outcomes were the proportion of patients who recovered based on the diagnosis and treatment, diagnostic modalities that contributed to the diagnosis, and factors (patients’ characteristics, symptoms, and laboratory data) related to the diagnosis or recovery.

### Statistical analysis

Continuous variables and categorical variables are shown as the mean ± standard deviation (SD) and number (percent), respectively. A logistic regression model was used to investigate the predictive factors for the presence or absence of diagnosis and recovery (diagnosis-providing factor and prognostic factor). Using the significant factors extracted using the univariate logistic regression analysis (P < 0.05), we performed multivariate logistic regression analysis and then extracted variables with a high predictive performance using the backward selection method (P < 0.05). We showed the minimum number of required factors using a variable selection method, based on the Occam's razor principle. In the logistic regression model, the odds ratios, 95% confidence intervals, and p-values (by a Wald test) were calculated. All data were analyzed with EZR 64-bit version (Saitama Medical Center, Jichi Medical University, Saitama, Japan) [22]. P < 0.05 was considered statistically significant.

### Ethics statement

To obtain data from the medical records, we disclosed the purpose and methods of this study to the study patients on the home-page of the Shizuoka General Hospital website and guaranteed their right to refuse participation in the study. When data on prognoses were additionally investigated by a telephone call, the purpose and methods were verbally explained to the participants or their families, and then we obtained individual consent. Identifiable information, such as individual names, etc., was deleted from the obtained data and only the anonymized data were used for this study. This study was approved by the clinical research ethics committee of Shizuoka General Hospital (approved on April 25, 2017; No. SGHIRB#2017006).

## Results

### Patients’ characteristics and prognoses

Of the 6,646 older adults aged ≥65 years who visited the emergency department during the study period, 243 had anorexia. Eighty-three patients met the inclusion criteria after the exclusion of 155 patients with underlying diseases, 4 patients who had two or more episodes of anorexia, and 1 patient who refused to undergo an additional investigation to determine the prognosis.

Data on the patient characteristics and prognoses are shown in Table 1. The mean age was 82.3 ± 8.3 years, and men comprised 50.6% of all patients. Seventy-one percent of the patients were hospitalized after the emergency department visit. The prognoses were as follows: recovered 78.3%, not changed 1.2%, and dead 20.5%.

**Table 1:** Patient characteristics and prognosis.

Variable	N = 83
Age (years), mean ± SD	82.3 ± 8.3
65–74	15 (18.1%)
75–84	29 (34.9%)
≥85	39 (47.0%)
Male	42 (50.6%)
Type of residence before the emergency department visit	
Home	73 (88.0%)
Facilities	9 (10.8%)
Others	1 (1.2%)
Ambulation	
Ambulatory	51 (61.5%)
Using cane or walker	16 (19.3%)
Wheelchair	3 (3.6%)
Bedridden	10 (12.1%)
Unknown	3 (3.6%)
Days from the onset to the visit (days), mean ± SD	14.5 ± 27.2
Hospitalized	59 (71.1%)
Length of hospitalization (days), mean ± SD	18.4 ± 15.5
Treated	51 (61.4%)
Prognosis	
Recovered	65 (78.3%)
Not changed	1 (1.2%)
Dead	17 (20.5%)

Values are presented as mean ± SD and n (%).

### Causes of anorexia

**Fig 1:**
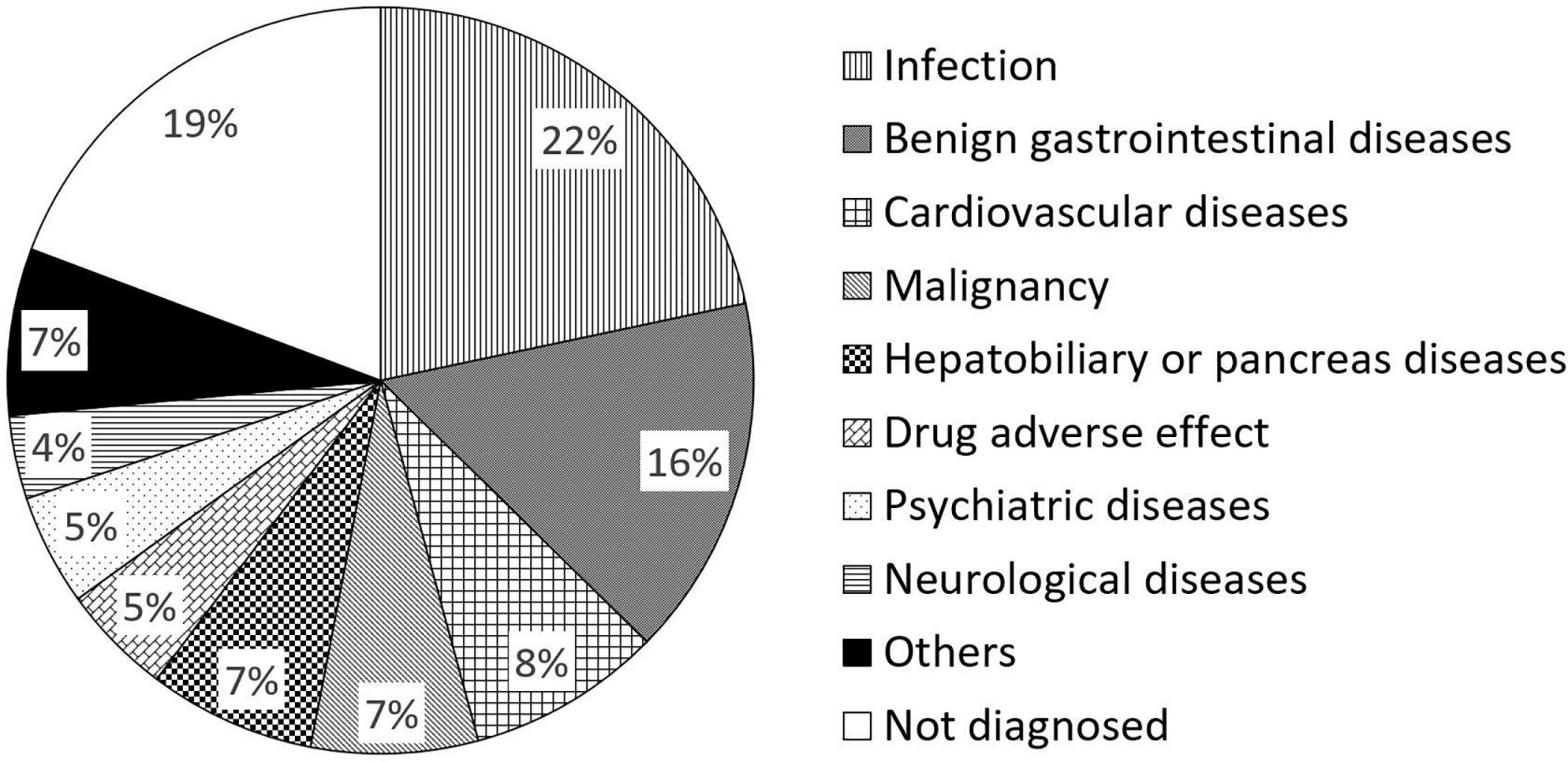
Causes of anorexia.

Fig 1 shows the causes of anorexia. In 67 patients (80.7%), the causes of anorexia were identified, and in 16 (19.3%), we could not reach a diagnosis despite examinations. The most frequent cause of anorexia was an infection (18 patients: including 9 with pneumonia, and 3 with urinary tract infection), followed by benign gastrointestinal diseases (13 patients: including 5 with peptic ulcer and 3 with ileus), and cardiovascular diseases (7 patients: including 3 with acute myocardial infarction and 2 with pulmonary embolism). Malignancy was observed in 6 patients (6.9%).

**Fig 2:**
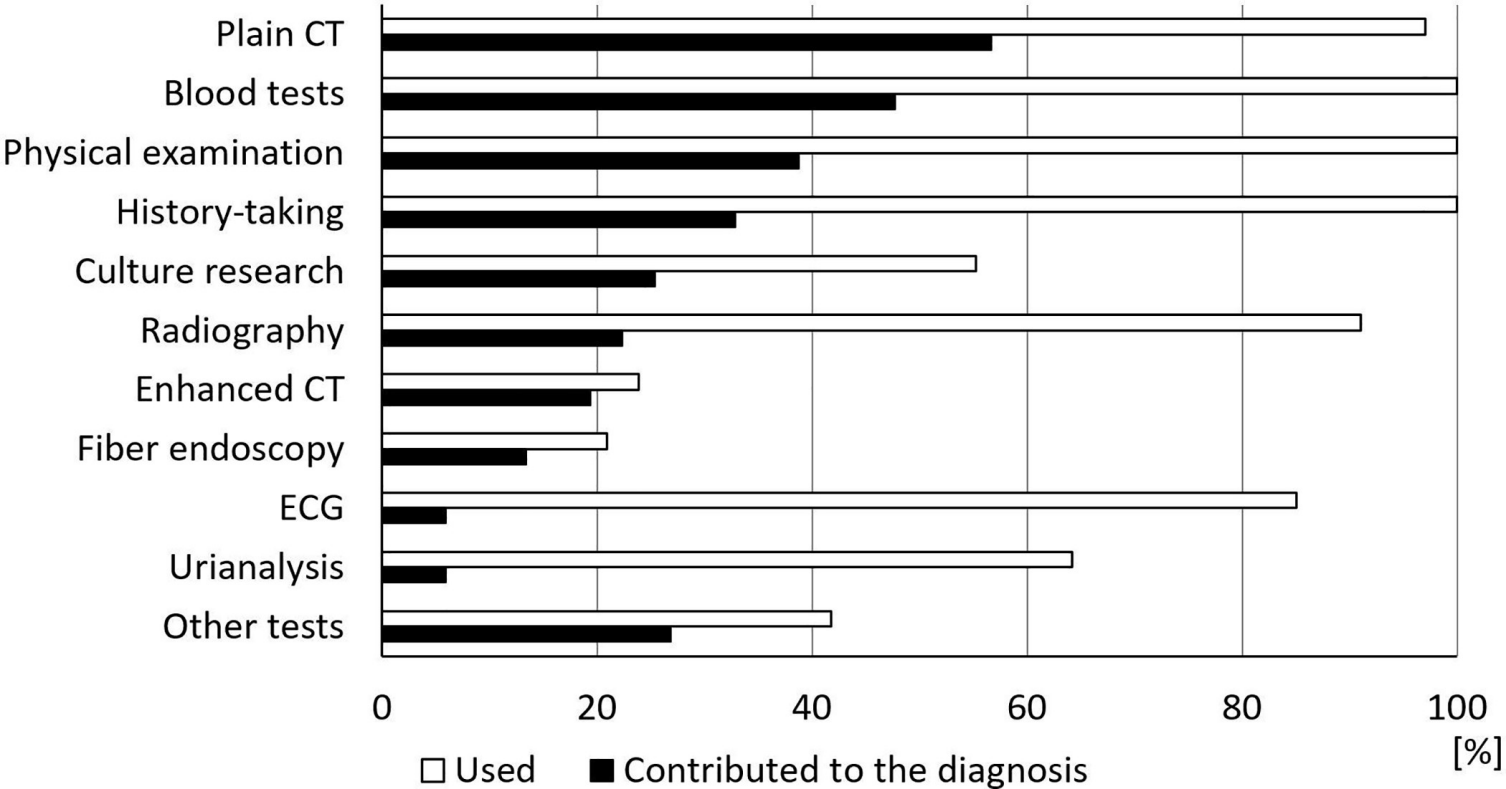
Diagnostic modalities. CT: computed tomography, ECG: electrocardiography.

Fig 2 shows the proportion of modalities that were used for and contributed to the diagnosis. The modality that most strongly contributed to the diagnosis was plain CT (56.7%) followed by blood tests (47.8%). Physical examinations (38.8%) and history-taking (32.8%) had less of a contribution. Plain CT contributed to the diagnosis in 38 patients, and the diagnoses were mainly pneumonia (8 patients), sepsis (3 patients), and ileus (3 patients). Plain CT was the only modality for diagnosis in 6 patients (including 2 patients with gastric cancer and 1 each with aspiration pneumonia, duodenitis, heart failure, and intraventricular brain hemorrhage).

### Treatment and recovery stratified by cause

Table 2 shows the proportions of treatment and recovery stratified by the causes of anorexia. Treatment was performed in 51 (76.1%) of the 67 patients with anorexia and known causes. While the treatment rate was low for psychiatric diseases (0%) and malignancy (16.7%), all of the patients with benign gastrointestinal diseases, cardiovascular diseases, and neurological diseases, and 94% of those with infections were treated.

**Table 2:** Proportion of treatment and recovery according to the cause of anorexia.

Cause	n	Treated (%)	Recovered (%)
All patients with known causes	67	51 (76.1%)	49 (73.1%)
Infection	18	17 (94.4%)	9 (50.0%)
Benign gastrointestinal diseases	13	13 (100%)	12 (92.3%)
Cardiovascular diseases	7	7 (100%)	4 (57.1%)
Malignancy	6	1 (16.7%)	4 (66.7%)
Benign hepatobiliary or pancreas diseases	6	3 (50.0%)	4 (66.7%)
Drug adverse effects	4	3 (75.0%)	4 (100%)
Psychiatric diseases	4	0 (0%)	3 (75.0%)
Neurological diseases	3	3 (100%)	3 (100%)
Others	6	4 (66.7%)	6 (100%)
Patients with unknown causes	16		16 (100%)

Of all 83 patients, 65 (78.3%) recovered their oral intake. Only 49 (73.1%) of the 67 patients with known causes recovered, while all 16 patients with unknown causes recovered. The proportions of patients with infections (50.0%) and cardiovascular disease (57.1%) who recovered were lower than that of the patients with malignancy (66.7%).

### Diagnosis-providing factor and prognostic factor

The results of univariate analyses for predicting the diagnosis and recovery are shown in S1 and S2 Tables, respectively. The results of the univariate and multivariate analysis for identified factors are shown in Table 3.

**Table 3:** Logistic regression analyses for predicting diagnosis and recovery.

Variables	Category (reference)	Univariate			Multivariate		
		Odds ratio	95% CI	p value	Odds ratio	95% CI	p value
Diagnosis							
WBC (×10^2^/μL)	1	1.02	1.00–1.04	0.014	1.03	1.01–1.06	0.018
TP (g/dL)	1	0.37	0.16–0.86	0.021	0.18	0.04–0.73	0.017
ALT (unit/L)	1	1.12	1.03–1.22	0.007	1.15	1.02–1.30	0.028
LDH (unit/L)	1	1.01	1.00–1.02	0.007	1.01	1.00–1.03	0.031
Recovery							
Unconsciousness	Y (vs. N)	0.07	0.02–0.25	<0.001	0.09	0.02–0.43	0.002
Alb (g/dL)	1	6.00	2.24–16.1	<0.001	4.70	1.52–14.50	0.007
T-bil (mg/dL)	1	0.42	0.22–0.78	0.006	0.38	0.17–0.83	0.015

Alb: albumin, ALT: alanine aminotransferase, CI: confidential interval, LDH: lactate dehydrogenase, T-bil: total bilirubin, TP: total protein, WBC: white blood cell

In the univariate analysis, the significant factors for predicting the diagnosis were the white blood cell count and levels of total protein, albumin, sodium, aspartate transaminase, alanine transaminase, lactate dehydrogenase, and C-reactive protein (S1 Table). Among them, the cause-providing factors were identified in multivariate analysis as the white blood cell count and the levels of total protein, alanine transaminase, and lactate dehydrogenase (Table 3).

The significant factors to predict recovery in univariate analysis were unconsciousness, as well as the levels of platelets, total protein, albumin, total bilirubin, blood urea nitrogen, sodium, and C-reactive protein (S2 Table). Among them, the factors to predict recovery were identified in multivariate analysis as unconsciousness and the levels of albumin and total bilirubin (Table 3).

## Discussion

Our study had three main findings. First, in 81% of the older adults with anorexia and no previously identified underlying diseases, the causes could be identified with the use of appropriate modalities. Second, the modality that most strongly contributed to diagnosis was plain CT followed by blood tests; the value of history-taking and physical examinations were limited. Third, only 73% of patients with a known cause recovered; in contrast, all patients with an unknown cause recovered.

For the causes of anorexia, previous studies reported that several factors, including physiological factors such as aging, as well as pathological and psychological factors, have complex associations in older adults [12, 13, 16, 19, 23]; however, few studies have evaluated the prevalence of the causes. Furthermore, our study population is different from previous studies. Anorexia was defined on the basis of the amount of food intake [24, 25] or results of the Simplified Nutritional Assessment Questionnaire (SNAQ) [26–28] in community-dwelling older adults [24, 26–30], long-term care residents [24, 26], or hospitalized patients [24, 25] in previous studies. In our study, anorexia was subjectively evaluated by patients or caregivers in older patients visiting the emergency department. Although measuring food intake precisely or evaluating by a questionnaire would not be suitable in the setting of emergency department (in fact, 15/83 patients [18%] of our study were unconscious), the evaluation of anorexia in our study might be somewhat inaccurate. Nevertheless, the present study showed that in most of the older adults with anorexia, the causes were identifiable, and the frequency of causes was revealed. In particular, older adults with anorexia and no apparent causes tend to be regarded as frailty and at the end of life [11]; however, if the diagnostic process is inadequate, there may be a risk of some identifiable causes being overlooked, leading to the chance of treatment being lost [30]. Evaluating nutritional status and amount of oral intake, and searching for a cause of anorexia should not be avoided simply because the patients are old.

For the diagnostic modality, history-taking and physical examinations were less helpful for the diagnosis than plain CT and blood tests. Previous studies on unintentional weight loss in older adults [31–36] showed that the cause was identified by thorough history-taking, targeted physical examinations, and simple laboratory tests in most cases; those results are different from the results obtained in this study. This difference could be attributed to that of the evaluated symptoms and study population. Anorexia of aging may lead to weight loss and cachexia [13, 19, 27, 30, 37, 38]. However, the time from onset of anorexia to visiting the emergency department was within 3 days in 27/83 patients (33%) of our study; this would not be sufficient to cause weight loss. However, the number of days from onset to visit did not correlate with the prediction of diagnosis nor recovery. Instead, the levels of total protein and albumin correlated to predicting diagnosis and recovery, respectively. The reason may be that the evaluation of anorexia was subjective and inaccurate in some patients. Similarly, Marton et al. [36] found that many people who claimed significant weight loss had not actually lost weight. Moreover, only 67% of the patients with a diagnosis of infection had a fever in our study, and there was no chest pain in the 3 patients diagnosed with acute myocardial infarction. The fact that the symptoms and physical findings in older adults can be vague or atypical may have limited the diagnostic value. Critical or treatable diseases should not be ruled out immediately even in patients with an atypical history or symptoms, but instead, low-invasive examinations should be considered proactively.

Regarding recovery, even though most patients with infection or cardiovascular diseases were treated, the proportion of those who recovered was lower than that of patients with malignancy. Diseases with therapeutic indications are not always curable; instead, end-of-life care is needed. Previous studies investigating heart failure in older adults reported that advanced age and comorbidities, such as renal impairment and anemia, were associated with all-cause death [9, 39]. Oshitani et al. [40] found that the prognosis of nursing- and healthcare-associated pneumonia was not related to whether antibiotic sensitive to causative bacteria was administered or not, but was related to a lower level of albumin. When treating older patients with anorexia, we should consider incorporating palliative care with advance care planning even if the causes are nonmalignant diseases [41, 42]. Additionally, all patients with unknown causes recovered in our study. Although these findings cannot be fully generalized because of the limited number of patients, patients with unknown causes showed lower levels of white blood cells, alanine transaminase, and lactate dehydrogenase, and a higher level of total protein. These results may indicate that they were less likely to have inflammatory diseases and hepatic dysfunction, and less malnourished. It is possible that older adults who were in a more severe condition and were not expected to recover may not have visited the emergency department of the tertiary care center and therefore were not included in the study. However, most of the anorexic patients with unknown causes could recover, especially if they are less malnourished. Therefore, such patients should not be regarded immediately as being at the end of life, but should instead be appropriately evaluated for their nutritional status and amount of oral intake.

This study has several limitations. First, as the sample size was small, some of the results, such as the proportions of treatment and recovery stratified by cause, were considerably affected by chance, and the number of patients with each concomitant symptom was also quite small. Younger patients with malignancy or organ failure visited our center more frequently than older adults without underlying diseases because the study was performed in a tertiary care center located in a relatively urban area. A higher number of older adults may have visited community-based medical facilities in areas with a high aging rate, and our findings would depend on different patient backgrounds. Second, we did not evaluate body weight, body mass index, or the amount of oral intake in either the recognition of anorexia or the recovery from anorexia. Therefore, it is difficult to determine whether the anorexia actually caused cachexia, and whether the recovery from anorexia was appropriately evaluated by the medical team. Further studies are needed to clarify the nutritional status and the amount of oral intake at the time of the visit and recovery of older patients with anorexia. Third, it was difficult to clarify the causal relationship between anorexia and some diseases that we regarded as the cause. For example, infection may not be the cause of anorexia but the result of decreased immunocompetence posed by anorexia and malnutrition [32]. In such cases, the “cause” should be rephrased to “likely explanation for the anorexia.” Further, the proportion of patients with psychiatric diseases was lower than that previously reported [31–36] because the studied hospital did not have a department of psychiatry. Only 4 patients were evaluated as having psychiatric diseases, and no patient received treatment. At least 2 patients were recommended to visit the department of psychiatry in another hospital; however, they chose not to do so. How to diagnose and treat psychiatric diseases appropriately in facilities without a department of psychiatry is an issue that requires further consideration. Finally, we deemed that our prediction models for cause and recovery were not valid because of a small sample size. For example, it was unclear in this study whether most of the concomitant symptoms or results of laboratory tests were associated with cause or recovery. However, a few exceptions indicated that patients having anorexia and unconsciousness concomitantly were predicted not to recover. Further studies investigating more elderly patients with concomitant symptoms are needed to identify how to predict cause and recovery of anorexia among older adults. In addition, more specific criteria may be needed to evaluate anorexia and concomitant symptoms.

In conclusion, in 81% of older adults with anorexia and no underlying diseases, the causes could be identified through the use of appropriate modalities, such as plain CT or blood tests, and while only three-quarters of patients with known causes recovered, all the patients with unknown causes recovered. Older adults with anorexia are not always at the end of life and efforts should be made to identify the causes, considering the limitations associated with treating infections and cardiovascular diseases in such patients.

## Supporting information

S1 Table Univariate logistic regression analysis for predicting diagnosis. Bolds indicate p < 0.05. Alb: albumin, ALT: alanine aminotransferase, APTT: activated partial thromboplastin time, AST: aspartate aminotransferase, BS: blood sugar, BUN: blood urea nitrogen, Ca: calcium, CI: confidential interval, CK: creatine kinase, Cl: chloride, Cr: creatinine, CRP: C reactive protein, D-bil: direct bilirubin, GGT: gamma-glutamyltransferase, Hb: hemoglobin, HDL-C: high-density lipoprotein cholesterol, K: potassium, LDH: lactate dehydrogenase, LDL-C: low-density lipoprotein cholesterol, Na: sodium, N/C: not converged, P: phosphate, Plt: platelet, PT: prothrombin time, RBC: red blood cell, SpO_2_: saturated oxygen, T-bil: total bilirubin, T-cho: total cholesterol, TG: triglyceride, TP: total protein, U-Glu: urine glucose, U-OB: urine occult blood, U-Pro: urine protein, WBC: white blood cell.
(DOCX)

S2 Table Univariate logistic regression analysis for predicting recovery. Bolds indicate p < 0.05. Alb: albumin, ALT: alanine aminotransferase, APTT: activated partial thromboplastin time, AST: aspartate aminotransferase, BS: blood sugar, BUN: blood urea nitrogen, Ca: calcium, CI: confidential interval, CK: creatine kinase, Cl: chloride, Cr: creatinine, CRP: C reactive protein, D-bil: direct bilirubin, GGT: gamma-glutamyltransferase, Hb: hemoglobin, HDL-C: high-density lipoprotein cholesterol, K: potassium, LDH: lactate dehydrogenase, LDL-C: low-density lipoprotein cholesterol, Na: sodium, N/C: not converged, P: phosphate, Plt: platelet, PT: prothrombin time, RBC: red blood cell, SpO_2_: saturated oxygen, T-bil: total bilirubin, T-cho: total cholesterol, TG: triglyceride, TP: total protein, U-Glu: urine glucose, U-OB: urine occult blood, U-Pro: urine protein, WBC: white blood cell.
(DOCX)
