# Supplementary material for: The cause of anorexia and proportion of its recovery in older adults without underlying disease: Results of a retrospective study
Source: PLoS One. 2019 Oct 24;14(10):e0224354. doi: 10.1371/journal.pone.0224354 (PMC6812872; doi:10.1371/journal.pone.0224354)
Supplement: S1 Table — Bolds indicate p < 0.05. Alb: albumin, ALT: alanine aminotransferase, APTT: activated partial thromboplastin time, AST: aspartate aminotransferase, BS: blood sugar, BUN: blood urea nitrogen, Ca: calcium, CI: confidential interval, CK: creatine kinase, Cl: chloride, Cr: creatinine, CRP: C reactive protein, D-bil: direct bilirubin, GGT: gamma-glutamyltransferase, Hb: hemoglobin, HDL-C: high-density lipoprotein cholesterol, K: potassium, LDH: lactate dehydrogenase, LDL-C: low-density lipoprotein cholesterol, Na: sodium, N/C: not converged, P: phosphate, Plt: platelet, PT: prothrombin time, RBC: red blood cell, SpO2: saturated oxygen, T-bil: total bilirubin, T-cho: total cholesterol, TG: triglyceride, TP: total protein, U-Glu: urine glucose, U-OB: urine occult blood, U-Pro: urine protein, WBC: white blood cell. (DOCX) [file pone.0224354.s001.docx]

**S1 Table. Univariate logistic regression analysis for predicting diagnosis**

| Variables | Category or unit | n | Diagnosed (n=70) | Not diagnosed (n=17) | Univariate logistic regression analysis | | |
| --- | --- | --- | --- | --- | --- | --- | --- |
|  |  |  | N (%) or mean ± SD | N (%) or mean ± SD | Odds ratio | 95% CI | p value |
| Age | 1 |  | 82.3 ± 8.6 | 82.1 ± 7.7 | 1.00 | 0.94–1.07 | 0.903 |
| Sex | Female | 83 | 34 (50.7%) | 7 (43.8%) | 1.00 | - |  |
|  | Male |  | 33 (49.3%) | 9 (56.3%) | 0.76 | 0.25–2.26 | 0.616 |
| Days from onset to the visit | 1 | 81 | 15.8 ± 30.1 | 9.2 ± 9.5 | 1.02 | 0.98–1.05 | 0.407 |
| Type of residence before the visit | Home | 82 | 58 (87.9%) | 15 (93. 8%) | 1.00 | - |  |
|  | Facility |  | 8 (12.1%) | 1 (6.3%) | 2.07 | 0.24–17.9 | 0.483 |
| Bedridden | N | 79 | 58 (89.2%) | 12 (85.7%) | 1.00 | - |  |
|  | Y |  | 7 (10.8%) | 2 (14.3%) | 0.71 | 0.13–3.86 | 0.693 |
| Weakness | N | 83 | 49 (73.1%) | 9 (56.3%) | 1.00 | - |  |
|  | Y |  | 18 (26.9%) | 7 (43.8%) | 0.47 | 0.15–1.46 | 0.192 |
| Malaise | N | 83 | 56 (83.6%) | 16 (100%) | 1.00 | - |  |
|  | Y |  | 11 (16.4%) | 0 (0%) | N/C | N/C | 0.993 |
| Walking difficulty | N | 83 | 62 (92.5%) | 13 (81.3%) | 1.00 | - |  |
|  | Y |  | 5 (7.5%) | 3 (18.8%) | 0.35 | 0.07–1.65 | 0.184 |
| Dizziness | N | 83 | 65 (97.0%) | 15 (93.8%) | 1.00 | - |  |
|  | Y |  | 2 (3.0%) | 1 (6.3%) | 0.46 | 0.04–5.43 | 0.539 |
| Thinness | N | 83 | 63 (94.0%) | 14 (87.5%) | 1.00 | - |  |
|  | Y |  | 4 (6.0%) | 2 (12.5%) | 0.44 | 0.07–2.67 | 0.375 |
| Low blood pressure | N | 83 | 63 (94.0%) | 15 (93.8%) | 1.00 | - |  |
|  | Y |  | 4 (6.0%) | 1 (6.3%) | 0.95 | 0.10–9.15 | 0.966 |
| Unconsciousness | N | 83 | 54 (80.6%) | 14 (87.5%) | 1.00 | - |  |
|  | Y |  | 13 (19.4%) | 2 (12.5%) | 1.69 | 0.34–8.35 | 0.523 |
| Fever | N | 83 | 58 (86.6%) | 15 (93.8%) | 1.00 | - |  |
|  | Y |  | 9 (13.4%) | 1 (6.3%) | 2.33 | 0.27 – 19.8 | 0.440 |
| Chills | N | 83 | 66 (98.5%) | 15 (93.8%) | 1.00 | - |  |
|  | Y |  | 1 (1.5%) | 1 (6.3%) | 0.23 | 0.01 – 3.84 | 0.304 |
| Low SpO_2_ | N | 83 | 65 (97.0%) | 15 (93.8%) | 1.00 | - |  |
|  | Y |  | 2 (3.0%) | 1 (6.3%) | 0.46 | 0.04–5.43 | 0.539 |
| Dyspnea | N | 83 | 63 (94.0%) | 15 (93.8%) | 1.00 | - |  |
|  | Y |  | 4 (6.0%) | 1 (6.3%) | 0.95 | 0.10–9.15 | 0.966 |
| Cough | N | 83 | 66 (98.5%) | 15 (93.8%) | 1.00 | - |  |
|  | Y |  | 1 (1.5%) | 1 (6.3%) | 0.23 | 0.01 – 3.84 | 0.304 |
| Dysphagia | N | 83 | 67 (100%) | 14 (87.5%) | 1.00 | - |  |
|  | Y |  | 0 (0%) | 2 (12.5%) | N/C | N/C | 0.991 |
| Abdominal pain | N | 83 | 65 (97.0%) | 16 (100%) | 1.00 | - |  |
|  | Y |  | 2 (3.0%) | 0 (0%) | N/C | N/C | 0.993 |
| Abdominal fullness | N | 83 | 61 (91.0%) | 16 (100%) | 1.00 | - |  |
|  | Y |  | 6 (9.0%) | 0 (0%) | N/C | N/C | 0.992 |
| Nausea/ vomiting | N | 83 | 50 (74.6%) | 15 (93.8%) | 1.00 | - |  |
|  | Y |  | 17 (25.4%) | 1 (6.3%) | 5.10 | 0.63–41.6 | 0.128 |
| Diarrhea | N | 83 | 63 (94.0%) | 15 (93.8%) | 1.00 | - |  |
|  | Y |  | 4 (6.0%) | 1 (6.3%) | 0.95 | 0.10–9.15 | 0.966 |
| Constipation | N | 83 | 65 (97.0%) | 15 (93.8%) | 1.00 | - |  |
|  | Y |  | 2 (3.0%) | 1 (6.3%) | 0.46 | 0.04–5.43 | 0.539 |
| Melena/ hematochezia | N | 83 | 64 (95.5%) | 16 (100%) | 1.00 | - |  |
|  | Y |  | 3 (4.5%) | 0 (0%) | N/C | N/C | 0.991 |
| Chest pain | N | 83 | 63 (94.0%) | 15 (93.8%) | 1.00 | - |  |
|  | Y |  | 4 (6.0%) | 1 (6.3%) | 0.95 | 0.10–9.15 | 0.966 |
| Back pain | N | 83 | 66 (98.5%) | 16 (100%) | 1.00 | - |  |
|  | Y |  | 1 (1.5%) | 0 (0%) | N/C | N/C | 0.992 |
| **WBC (×10^2^/μL)** | **1** | **83** | **111.5 ± 49.0** | **78.2 ± 28.0** | **1.02** | **1.00**–**1.04** | **0.014** |
| RBC (×10^4^/μL) | 1 | 83 | 394.1 ± 83.1 | 397.3 ± 74.4 | 1.00 | 0.99–1.01 | 0.887 |
| Hb (g/dL) | 1 | 83 | 11.9 ± 2.7 | 12.2 ± 2.1 | 0.97 | 0.78–1.20 | 0.758 |
| Plt (×10^4^/μL) | 1 | 83 | 23.1 ± 10.4 | 26.4 ± 9.3 | 0.97 | 0.92–1.02 | 0.247 |
| PT (second) | 1 | 68 | 13.8 ± 2.9 | 14.8 ± 5.8 | 0.93 | 0.80–1.09 | 0.371 |
| APTT (second) | 1 | 67 | 28.3 ± 6.9 | 28.7 ± 6.4 | 1.00 | 0.91–1.09 | 0.859 |
| **TP (g/dL)** | **1** | **80** | **6.9 ± 0.8** | **7.5 ± 0.6** | **0.37** | **0.16–0.86** | **0.021** |
| **Alb (g/dL)** | **1** | **83** | **3.2 ± 0.7** | **3.7 ± 0.6** | **0.28** | **0.10–0.78** | **0.015** |
| T-bil (mg/dL) | 1 | 83 | 1.3 ± 0.9 | 0.8 ± 0.4 | 3.33 | 0.97–11.5 | 0.057 |
| D-bil (mg/dL) | 1 | 27 | 0.6 ± 0.9 | 0.1 ± 0.1 | N/C | N/C | 0.103 |
| T-cho (mg/dL) | 1 | 11 | 154.7 ± 36.1 | 203.5 ± 19.1 | 0.94 | 0.86–1.02 | 0.137 |
| HDL-C (mg/dL) | 1 | 10 | 35.0 ± 17.1 | 35.0 ± 0.0 | 1.00 | 0.87–1.14 | 1.000 |
| LDL-C (mg/dL) | 1 | 6 | 90.2 ± 38.7 | 138.0 ± 0.0 | 0.96 | 0.88–1.05 | 0.330 |
| TG (mg/dL) | 1 | 12 | 127.2 ± 89.7 | 124.0 ± 31.1 | 1.00 | 0.98–1.02 | 0.958 |
| BUN (mg/dL) | 1 | 83 | 39.6 ± 30.1 | 37.8 ± 30.9 | 1.00 | 0.98–1.02 | 0.829 |
| Cr (mg/dL) | 1 | 83 | 1.4 ± 1.3 | 1.6 ± 1.5 | 0.90 | 0.62–1.30 | 0.578 |
| **Na (mmol/L)** | **1** | **83** | **138.4 ± 6.9** | **133.4 ± 10.1** | **1.08** | **1.01–1.17** | **0.028** |
| K (mmol/L) | 1 | 83 | 4.2 ± 0.7 | 4.2 ± 0.7 | 1.04 | 0.48–2.26 | 0.930 |
| Cl (mmol/L) | 1 | 83 | 100.1 ± 13.2 | 97.9 ± 10.3 | 1.01 | 0.98–1.05 | 0.530 |
| Ca (mg/dL) | 1 | 68 | 8.8 ± 0.7 | 9.0 ± 0.5 | 0.63 | 0.21 – 1.96 | 0.429 |
| P (mg/dL) | 1 | 51 | 4.2 ± 2.0 | 3.4 ± 1.7 | 1.31 | 0.75–2.29 | 0.336 |
| **AST (unit/L)** | **1** | **83** | **130.9 ± 357.5** | **23.1 ± 8.0** | **1.07** | **1.01**–**1.14** | **0.018** |
| **ALT (unit/L)** | **1** | **83** | **82.5 ± 270.7** | **13.1 ± 7.2** | **1.12** | **1.03–1.22** | **0.007** |
| **LDH (unit/L)** | **1** | **80** | **480.5 ± 1117.1** | **216.6 ± 61.4** | **1.01** | **1.00–1.02** | **0.007** |
| CK (unit/L) | 1 | 81 | 395.4 ± 908.6 | 112.6 ± 77.2 | 1.00 | 1.00–1.01 | 0.157 |
| GGT (unit/L) | 1 | 62 | 76.9 ± 99.4 | 24.0 ± 10.8 | 1.02 | 0.99–1.05 | 0.145 |
| **CRP (mg/dL)** | **1** | **83** | **6.9 ± 8.7** | **1.1 ± 1.8** | **1.40** | **1.00–1.97** | **0.047** |
| BS (mg/dL) | 1 | 66 | 146.9 ± 60.5 | 160.6 ± 111.2 | 1.00 | 0.99–1.01 | 0.552 |
| U-Pro | N | 47 | 14 (35.9%) | 4 (50.0%) | 1.00 | - |  |
|  | Y |  | 25 (64.1%) | 4 (50.0%) | 1.79 | 0.39–8.27 | 0.458 |
| U-Glu | N | 47 | 32 (82.1%) | 6 (75.0%) | 1.00 | - |  |
|  | Y |  | 7 (17.9%) | 2 (25.0%) | 0.66 | 0.11–3.96 | 0.646 |
| U-OB | N | 47 | 22 (56.4%) | 4 (50.0%) | 1.00 | - |  |
|  | Y |  | 17 (43.6%) | 4 (50.0%) | 0.77 | 0.17–3.55 | 0.740 |

Bolds indicate p < 0.05.

Alb: albumin, ALT: alanine aminotransferase, APTT: activated partial thromboplastin time, AST: aspartate aminotransferase, BS: blood sugar, BUN: blood urea nitrogen, Ca: calcium, CI: confidential interval, CK: creatine kinase, Cl: chloride, Cr: creatinine, CRP: C reactive protein, D-bil: direct bilirubin, GGT: gamma-glutamyltransferase, Hb: hemoglobin, HDL-C: high-density lipoprotein cholesterol, K: potassium, LDH: lactate dehydrogenase, LDL-C: low-density lipoprotein cholesterol, Na: sodium, N/C: not converged, P: phosphate, Plt: platelet, PT: prothrombin time, RBC: red blood cell, SpO_2_: saturated oxygen, T-bil: total bilirubin, T-cho: total cholesterol, TG: triglyceride, TP: total protein, U-Glu: urine glucose, U-OB: urine occult blood, U-Pro: urine protein, WBC: white blood cell
