# Supplementary material for: The cause of anorexia and proportion of its recovery in older adults without underlying disease: Results of a retrospective study
Source: PLoS One. 2019 Oct 24;14(10):e0224354. doi: 10.1371/journal.pone.0224354 (PMC6812872; doi:10.1371/journal.pone.0224354)
Supplement: S2 Table — Bolds indicate p < 0.05. Alb: albumin, ALT: alanine aminotransferase, APTT: activated partial thromboplastin time, AST: aspartate aminotransferase, BS: blood sugar, BUN: blood urea nitrogen, Ca: calcium, CI: confidential interval, CK: creatine kinase, Cl: chloride, Cr: creatinine, CRP: C reactive protein, D-bil: direct bilirubin, GGT: gamma-glutamyltransferase, Hb: hemoglobin, HDL-C: high-density lipoprotein cholesterol, K: potassium, LDH: lactate dehydrogenase, LDL-C: low-density lipoprotein cholesterol, Na: sodium, N/C: not converged, P: phosphate, Plt: platelet, PT: prothrombin time, RBC: red blood cell, SpO2: saturated oxygen, T-bil: total bilirubin, T-cho: total cholesterol, TG: triglyceride, TP: total protein, U-Glu: urine glucose, U-OB: urine occult blood, U-Pro: urine protein, WBC: white blood cell. (DOCX) [file pone.0224354.s002.docx]

**S2 Table. Univariate logistic regression analysis for predicting recovery**

| Variables | Category or unit | N | Recovered (n=69) | Not recovered (n=18) | Univariate logistic regression analysis | | |
| --- | --- | --- | --- | --- | --- | --- | --- |
|  |  |  | N (%) or mean ± SD | N (%) or mean ± SD | Odds ratio | 95% CI | p value |
| Age | 1 | 83 | 81.7 ± 7.8 | 84.4 ± 10.1 | 0.96 | 0.90–1.03 | 0.218 |
| Sex | Female | 83 | 35 (53.8%) | 6 (33.3%) | 1.00 | - |  |
|  | Male |  | 30 (46.2%) | 12 (66.7%) | 0.43 | 0.14–1.28 | 0.129 |
| Days from onset to the visit | 1 | 81 | 12.3 ± 26.6 | 22.2 ± 29.5 | 0.99 | 0.97–1.01 | 0.209 |
| Type of residence before the visit | Home | 82 | 57 (89.1%) | 16 (88.9%) | 1.00 | - |  |
|  | Facility |  | 7 (10.9%) | 2 (11.1%) | 0.98 | 0.19–5.21 | 0.983 |
| Bedridden | N | 82 | 55 (88.7%) | 16 (88.9%) | 1.00 |  |  |
|  | Y |  | 7 (11.3%) | 2 (11.1%) | 1.02 | 0.19–5.39 | 0.983 |
| Hospitalization | N | 83 | 21 (32.3%) | 3 (16.7%) | 1.00 | - |  |
|  | Y |  | 44 (67.7%) | 15 (83.3%) | 0.42 | 0.11–1.61 | 0.205 |
| Treatment | N | 83 | 27 (41.5%) | 13 (72.2%) | 1.00 | - |  |
|  | Y |  | 38 (58.5%) | 5 (27.8%) | 0.54 | 0.17–1.70 | 0.293 |
| Weakness | N | 83 | 44 (67.7%) | 14 (77.8%) | 1.00 | - |  |
|  | Y |  | 21 (32.3%) | 4 (22.2%) | 1.67 | 0.49–5.70 | 0.412 |
| Malaise | N | 83 | 54 (83.1%) | 18 (100%) | 1.00 | - |  |
|  | Y |  | 11 (16.9%) | 0 (0%) | N/C | N/C | 0.993 |
| Walking difficulty | N | 83 | 58 (89.2%) | 17 (94.4%) | 1.00 | - |  |
|  | Y |  | 7 (10.8%) | 1 (5.6%) | 2.05 | 0.24–17.9 | 0.515 |
| Dizziness | N | 83 | 63 (96.9%) | 17 (94.4%) | 1.00 | - |  |
|  | Y |  | 2 (3.1%) | 1 (5.6%) | 0.54 | 0.05–6.31 | 0.623 |
| Thinness | N | 83 | 61 (93.8%) | 16 (88.9%) | 1.00 | - |  |
|  | Y |  | 4 (6.2%) | 2 (11.1%) | 0.53 | 0.09–3.12 | 0.479 |
| Low blood pressure | N | 83 | 62 (95.4%) | 16 (88.9%) | 1.00 | - |  |
|  | Y |  | 3 (4.6%) | 2 (11.1%) | 0.39 | 0.06–2.52 | 0.320 |
| **Unconsciousness** | **N** | **83** | **60 (92.3%)** | **8 (44.4%)** | **1.00** | **-** |  |
|  | **Y** |  | **5 (7.7%)** | **10 (55.6%)** | **0.07** | **0.02**–**0.25** | **<0.001** |
| Fever | N | 83 | 56 (86.2%) | 17 (94.4%) | 1.00 | - |  |
|  | Y |  | 9 (13.8%) | 1 (5.6%) | 2.73 | 0.32–23.1 | 0.356 |
| Chill | N | 83 | 63 (96.9%) | 18 (100%) | 1.00 | - |  |
|  | Y |  | 2 (3.1%) | 0 (0%) | N/C | N/C | 0.993 |
| Low SpO_2_ | N | 83 | 63 (96.9%) | 17 (94.4%) | 1.00 | - |  |
|  | Y |  | 2 (3.1%) | 1 (5.6%) | 0.54 | 0.05–6.31 | 0.623 |
| Dyspnea | N | 83 | 60 (92.3%) | 18 (100%) | 1.00 | - |  |
|  | Y |  | 5 (7.7%) | 0 (0%) | N/C | N/C | 0.993 |
| Cough | N | 83 | 63 (96.9%) | 18 (100%) | 1.00 | - |  |
|  | Y |  | 2 (3.1%) | 0 (0%) | N/C | N/C | 0.993 |
| Dysphagia | N | 83 | 63 (96.9%) | 18 (100%) | 1.00 | - |  |
|  | Y |  | 2 (3.1%) | 0 (0%) | N/C | N/C | 0.993 |
| Abdominal pain | N | 83 | 63 (96.9%) | 18 (100%) | 1.00 | - |  |
|  | Y |  | 2 (3.1%) | 0 (0%) | N/C | N/C | 0.993 |
| Abdominal fullness | N | 83 | 61 (93.8%) | 16 (88.9%) | 1.00 | - |  |
|  | Y |  | 4 (6.2%) | 2 (11.1%) | 0.53 | 0.09–3.12 | 0.479 |
| Nausea/ Vomiting | N | 83 | 47 (72.3%) | 18 (100%) | 1.00 | - |  |
|  | Y |  | 18 (27.7%) | 0 (0%) | N/C | N/C | 0.991 |
| Diarrhea | N | 83 | 60 (92.3%) | 18 (100%) | 1.00 | - |  |
|  | Y |  | 5 (7.7%) | 0 (0%) | N/C | N/C | 0.993 |
| Constipation | N | 83 | 62 (95.4%) | 18 (100%) | 1.00 | - |  |
|  | Y |  | 3 (4.6%) | 0 (0%) | N/C | N/C | 0.991 |
| Melena/ hematochezia | N | 83 | 62 (95.4%) | 18 (100%) | 1.00 | - |  |
|  | Y |  | 3 (4.6%) | 0 (0%) | N/C | N/C | 0.991 |
| Chest pain | N | 83 | 60 (92.3%) | 18 (100%) | 1.00 | - |  |
|  | Y |  | 5 (7.7%) | 0 (0%) | N/C | N/C | 0.993 |
| Back pain | N | 83 | 64 (98.5%) | 18 (100%) | 1.00 | - |  |
|  | Y |  | 1 (1.5%) | 0 (0%) | N/C | N/C | 0.992 |
| WBC (×10^2^/μL) | 1 | 83 | 102.5 ± 40.7 | 114.5 ± 66.9 | 1.00 | 0.98–1.01 | 0.343 |
| RBC (×10^4^/μL) | 1 | 83 | 399.0 ± 81.1 | 378.9 ± 81.3 | 1.00 | 1.00–1.01 | 0.352 |
| Hb (g/dL) | 1 | 83 | 12.1 ± 2.6 | 11.4 ± 2.6 | 1.12 | 0.91–1.36 | 0.280 |
| **Plt (×10^4^/μL)** | **1** | **83** | **25.2 ± 10.3** | **18.4 ± 8.4** | **1.09** | **1.02–1.17** | **0.016** |
| PT (s) | 1 | 68 | 13.5 ± 3.1 | 15.0 ± 4.2 | 0.90 | 0.77–1.04 | 0.160 |
| APTT (s) | 1 | 67 | 27.3 ± 4.1 | 31.3 ± 11.0 | 0.92 | 0.84–1.00 | 0.064 |
| **TP (g/dL)** | **1** | **80** | **7.2 ± 0.8** | **6.5 ± 0.8** | **3.00** | **1.45–6.23** | **0.003** |
| **Alb (g/dL)** | **1** | **83** | **3.5 ± 0.6** | **2.8 ± 0.7** | **6.00** | **2.24–16.1** | **<0.001** |
| **T-bil (mg/dL)** | **1** | **83** | **1.0 ± 0.7** | **1.7 ± 1.1** | **0.42** | **0.22–0.78** | **0.006** |
| D-bil (mg/dL) | 1 | 27 | 0.4 ± 0.6 | 1.0 ± 1.1 | 0.38 | 0.12–1.18 | 0.094 |
| T-cho (mg/dL) | 1 | 11 | 164.3 ± 45.6 | 161.7 ± 8.5 | 1.00 | 0.97–1.04 | 0.917 |
| HDL-C (mg/dL) | 1 | 10 | 38.0 ± 16.5 | 30.5 ± 16.8 | 1.04 | 0.94–1.14 | 0.460 |
| LDL-C (mg/dL) | 1 | 6 | 98.8 ± 44.4 | 95.0 ± 0.0 | 1.00 | 0.94–1.07 | 0.924 |
| TG (mg/dL) | 1 | 12 | 95.9 ± 36.3 | 219.0 ± 119.8 | 0.98 | 0.94–1.01 | 0.112 |
| **BUN (mg/dL)** | **1** | **83** | **35.5 ± 30.8** | **52.8 ± 23.2** | **0.98** | **0.97–1.00** | **0.042** |
| Cr (mg/dL) | 1 | 83 | 1.4 ± 1.5 | 1.6 ± 0.9 | 0.88 | 0.62–1.24 | 0.458 |
| **Na (mmol/L)** | **1** | **83** | **136.2 ± 7.4** | **141.6 ± 8.2** | **0.90** | **0.82–0.98** | **0.016** |
| K (mmol/L) | 1 | 83 | 4.1 ± 0.7 | 4.3 ± 0.8 | 0.80 | 0.39–1.67 | 0.558 |
| Cl (mmol/L) | 1 | 83 | 98.9 ± 13.3 | 102.6 ± 9.6 | 0.96 | 0.89–1.03 | 0.237 |
| Ca (mg/dL) | 1 | 68 | 8.9 ± 0.6 | 8.7 ± 0.8 | 1.99 | 0.85–4.67 | 0.115 |
| P (mg/dL) | 1 | 51 | 3.8 ± 1.9 | 4.8 ± 2.1 | 0.79 | 0.58–1.06 | 0.112 |
| AST (unit/L) | 1 | 83 | 99.1 ± 340.8 | 150.0 ± 256.0 | 1.00 | 1.00–1.00 | 0.562 |
| ALT (unit/L) | 1 | 83 | 72.1 ± 275.2 | 58.3 ± 55.2 | 1.00 | 1.00–1.00 | 0.832 |
| LDH (unit/L) | 1 | 80 | 410.7 ± 1103.5 | 486.4 ± 553.3 | 1.00 | 1.00–1.00 | 0.779 |
| CK (unit/L) | 1 | 81 | 289.5 ± 848.8 | 514.6 ± 710.0 | 1.00 | 1.00–1.00 | 0.343 |
| GGT (unit/L) | 1 | 62 | 55.5 ± 82.3 | 97.5 ± 110.4 | 1.00 | 0.99–1.00 | 0.139 |
| **CRP (mg/dL)** | **1** | **83** | **4.7 ± 7.0** | **9.7 ± 10.7** | **0.94** | **0.88–0.99** | **0.031** |
| BS (mg/dL) | 1 | 66 | 143.8 ± 60.3 | 166.9 ± 98.9 | 1.00 | 0.99–1.00 | 0.276 |
| U-Pro | N | 47 | 14 (43.8%) | 12 (75.0%) | 1.00 | - |  |
|  | Y |  | 17 (56.3%) | 4 (25.0%) | 0.41 | 0.11–1.54 | 0.184 |
| U-Glu | N | 47 | 25 (81.3%) | 13 (81.3%) | 1.00 | - |  |
|  | Y |  | 6 (18.8%) | 3 (18.8%) | 1.04 | 0.22–4.85 | 0.960 |
| U-OB | N | 47 | 17 (53.1%) | 9 (56.3%) | 1.00 | - |  |
|  | Y |  | 14 (46.9%) | 7 (43.8%) | 1.06 | 0.31–3.57 | 0.927 |

Bolds indicate p < 0.05.

Alb: albumin, ALT: alanine aminotransferase, APTT: activated partial thromboplastin time, AST: aspartate aminotransferase, BS: blood sugar, BUN: blood urea nitrogen, Ca: calcium, CI: confidential interval, CK: creatine kinase, Cl: chloride, Cr: creatinine, CRP: C reactive protein, D-bil: direct bilirubin, GGT: gamma-glutamyltransferase, Hb: hemoglobin, HDL-C: high-density lipoprotein cholesterol, K: potassium, LDH: lactate dehydrogenase, LDL-C: low-density lipoprotein cholesterol, Na: sodium, N/C: not converged, P: phosphate, Plt: platelet, PT: prothrombin time, RBC: red blood cell, SpO_2_: saturated oxygen, T-bil: total bilirubin, T-cho: total cholesterol, TG: triglyceride, TP: total protein, U-Glu: urine glucose, U-OB: urine occult blood, U-Pro: urine protein, WBC: white blood cell
